# Head Turning-Induced Hypotension in Elderly People

**DOI:** 10.1371/journal.pone.0072837

**Published:** 2013-08-16

**Authors:** Yvonne Schoon, Marcel G. M. Olde Rikkert, Sara Rongen, Joep Lagro, Bianca Schalk, Jurgen A. H. R. Claassen

**Affiliations:** Department of Geriatric Medicine Radboud University Nijmegen Medical Centre, Nijmegen, The Netherlands; University of Düsseldorf, Germany

## Abstract

Carotid sinus hypersensitivity has a high prevalence in the elderly and is a possible cause of falls. In carotid sinus hypersensitivity, external triggers cause sudden reductions in blood pressure, leading to dizziness or syncope, resulting in falls. Turning of the head is considered an important example of such an external trigger in everyday life, wherein rotation of the neck is thought to manipulate the hypersensitive carotid sinus. However, direct evidence for this is lacking. The aim of this study was to investigate the effects of head turning in elderly with carotid sinus hypersensitivity. We performed a prospective, observational study in 105 elderly patients who visited a geriatric falls clinic in a university teaching hospital and in 25 community dwelling healthy elderly subjects. Continuous measurements of blood pressure and heart rate (Finapres) were performed before, during, and after head turning. Head turning-induced hypotension was defined as a drop in systolic blood pressure of at least 20 mmHg during head turning. Carotid sinus hypersensitivity was examined with carotid sinus massage. We also tested for two other common geriatric hypotensive syndromes, orthostatic hypotension and post prandial hypotension, using active standing and a meal test. All three hypotensive syndromes were defined using consensus definitions. Head turning resulted in hypotension in 39% of patients (mean systolic blood pressure drop 36 mm Hg) and in 44% of the healthy elderly, irrespective of the direction of the head movement. Carotid sinus hypersensitivity was associated with head-turning induced hypotension (OR= 3.5, 95% CI= 1.48 to 8.35). We conclude that head turning is indeed an important cause of sudden drops in blood pressure in elderly with carotid sinus hypersensitivity.

## Introduction

Falls are common in elderly people, 30% of those aged 65 years and older fall at least once a year [1]; [2]. The causal pathway of falling is generally multifactorial [3]. Guidelines recommend that primary care physicians screen for fall risks in all elderly people and that a comprehensive geriatric fall risk assessment be performed in those patients who have fallen [4].

An important aspect of this multifactorial screening process is the evaluation of blood pressure instability. The elderly are prone to the so-called hypotensive syndromes (e.g. orthostatic and post prandial hypotension). During hypotensive episodes, cerebral blood flow is temporarily reduced, which may manifest as dizziness or syncope. Through this mechanism, hypotensive syndromes contribute importantly to falls in the elderly [5]; [6].

Among these hypotensive syndromes, carotid sinus hypersensitivity (CSH) is thought to be an especially relevant contributing or causal factor in unexplained falls [6]; [7]. In CSH, the baroreceptors in the carotid sinus are thought to be overly irritable (e.g. to external manipulation or pressure) and as a result falsely register hypertension, leading to baroreflex-mediated rapid reduction in blood pressure. Although carotid sinus irritability [8] is a more appropriate term, herein we use CSH as this is the most commonly used term.

In elderly with CSH, mechanical manipulation of the carotid, as may occur when wearing a tight collar or during shaving, may cause a reduction in blood pressure and/or heart rate resulting in syncope and falls [9]; [10].

An additional and even more common trigger may be turning of the head. In case reports, clinically significant drops in blood pressure (sufficient to be considered a plausible cause of syncope) have been demonstrated when subjects turned their heads [11]. This observation could explain the relationship between CSH and falls [6]; [7], as this hypotensive response to head turning would occur frequently in everyday life.

The objective of this study was therefore to investigate the effect of head turning in elderly with CSH. We have tested the hypothesis that head turning triggers hypotensive episodes in elderly with CSH. We have investigated this primarily in a population where this information has the highest clinical relevance, i.e in elderly with a history of falls. In addition, to further explore the effect of head turning on blood pressure, we also included a group of healthy elderly, where, as indicated above, CSH is also prevalent.

## Methods

### Ethics Statement

The test protocol explained below was part of the standard care for patients who visited the Geriatric Falls Clinic and conformed to the Declaration of Helsinki, informed consent was asked and obtained for the tests. All healthy participants signed informed consent forms. The ethical review board (Commissie Mensgebonden Onderzoek region Arnhem-Nijmegen) approved the study.

### Study Population

This prospective, observational study was carried out in the Geriatric Falls Clinic in a university teaching hospital. Between March 2006 and November 2008, all ambulatory patients older than 65 years were recruited who had been referred for falls, dizziness and/or syncope. Patients were excluded when they met any exclusion criterion for carotid sinus massage or when hospital admission was required.

Functional performance was evaluated with the Groningen Activity Restriction Scale (GARS) [12]; [13]. Illness burden and diversity were estimated using the Cumulative Illness Rating Scale for Geriatrics (CIRS-G) [14]. Patients were asked whether a fall or dizziness was ever preceded by shaving or head turning. This question was used to assess the relationship between the results of the head turning test and each patient’s fall history. In each patient, the physician stated, before the Finapres test protocol was performed, if he expected the presence of a hypotensive syndrome contributing to falls.

In addition to this patient sample, a group of healthy elderly volunteers was recruited from the community. Their health status was examined including their medical history, drug use, functional performance, and a physical examination was performed. This group had no fall history.

### Blood Pressure Measurements

A ‘clinic’ blood pressure was recorded during the outpatient clinical evaluation using a sphygmomanometry device. During the test protocol, executed on a different day, beat-to-beat blood pressure and heart rate were continuously measured from the finger using the photoplethysmographic method (Finapres, Finapres Medical Systems, Amsterdam). During the test protocol, ECG registration was used for safety reasons. The Finapres method is a non-invasive technique that measures beat-to-beat variations in blood pressure and heart rate that has been validated against intra-arterial recordings as well as conventional sphygmomanometry [15]. After an overnight fast, medications were stopped from 6pm the day before testing until completion of the blood pressure measurements.

#### Head-turning test

The Head turning test (HTT) was performed in all subjects that underwent the Finapres test protocol. After at least ten minutes of active standing (until blood pressure was stable for at least five minutes), each subject was asked to perform three different head movements during 10-15 seconds: rotation to the right, rotation to the left and hyperextension. Between the different head movements, the head was at ‘rest’ in a normal position (facing forward) until the blood pressure was again stable for at least one minute. Systolic blood pressure and heart rate before the HTT were calculated as the mean of the ten beats preceding HTT. For each head movement separately, the systolic blood pressure and heart rate during the HTT were calculated as the mean of the three beats with the lowest systolic blood pressure. A positive result was defined as a drop in systolic blood pressure of at least 20 mmHg during one or more of the head movements and was called ‘head turning-induced hypotension’ (HTIH). We considered that changes in systolic blood pressure less than 20 mmHg can fall within the normal range of blood pressure fluctuations [16], and therefore to attribute these changes to head turning would promote false-positive association. This cut-off of 20 mmHg also complied with the customary clinical cut-offs used to define orthostatic and postprandial hypotension.

#### Test for hypotensive syndromes (including CSH)

The Finapres test protocol further consisted of a carotid sinus massage to assess CSH, an active standing test to evaluate orthostatic hypotension (OH), and a meal test to evaluate postprandial hypotension (PPH). During the tests, the subjects were asked whether they experienced symptoms.

Patients with known significant carotid stenosis, a (recent) history of ventricular arrhythmia, myocardial infarction or cerebral ischemia were excluded from carotid sinus (CS) massage [9]. During the CS massage procedure, the subjects laid on a tilt table. After five minutes of rest, the right carotid artery sinus was massaged for five seconds. One minute after normalisation of blood pressure or, in case symptoms were present, return to a normal state, the left carotid artery sinus was massaged. In cases yielding negative results, the test was repeated with the subject in a 70^0^-tilt position (CSH in tilt). CSH was defined as an R-R interval of at least three seconds (cardio-inhibitory type), a decrease of systolic blood pressure of 50 mmHg or more (vasodepressor type), or a combination of both (mixed type), irrespective of whether the subject was in a supine or tilted position [17].

After a ten-minute rest period in the supine position, the subjects were asked to stand up and to remain standing for ten minutes. OH was defined as a decrease of at least 20 mmHg in systolic blood pressure recorded from any of the ten-minute averages following the first 30 seconds after rising [18].

After a ten-minute rest period, the subjects consumed a standardised fluid meal within ten minutes. This meal consisted of 100 ml of glucose syrup (Nutrical®) and 100 ml of lactose-free whole milk (Soy milk calcium Alpro®) with a total of 292 calories, containing two grams of fat and four grams of protein. Heart rate and blood pressure were continuously measured from ten minutes before the start of the meal until 75 minutes after the meal. PPH was defined as a decrease in systolic blood pressure of 20 mmHg or more during any of these intervals [19].

The exact order of the test protocol was as follows; OH test, HTT, PPH test and CS massage.

CS massage was performed 80 minutes after the start of the meal test and when blood pressure had fully restored from any PPH, to ascertain that PPH did not influence assessment of CSH.

The baroreflex index was calculated by dividing the maximal change in heart rate by the maximal change in systolic blood pressure within the first 30 seconds after standing.

### Statistical Analyses

We used the same cut-off value for HTT as was used for OH and PPH (a drop in systolic blood pressure of 20 mmHg). Because this cut-off value yielded relatively mild blood pressure reductions, we performed an additional analysis with a cut-off of 30 mmHg for HTT.

The baseline characteristics of the participants, according to HTIH status (positive/negative, regardless which or how many positive head movements were present), were compared using t-tests for continuous variables and Pearson’s chi-square test for categorical variables. Two-tailed P-values of less than 0.05 were considered significant. Each hypotensive syndrome was considered as present or absent (dichotomous variable) in the analysis. Logistic regression analysis was used to examine the association between HTIH status (dependent variable) and CSH. The results were presented as odds ratio with a 95% confidence interval (CI). The following factors were included in the model for adjustment: age, sex, body mass index and systolic blood pressure. Because of the small size of the healthy group, only descriptive characteristics were reported there, besides a comparison of HTIH status between the patients and healthy elderly, using the Pearson’s chi-square test. All data processing and analyses were done with SPSS, version 16.0.01 (SPSS, Chicago, Illinois).

## Results

During the study period, 208 patients aged over 65 years visited the Geriatric Falls Clinic. In total, 105 patients met the enrolment criteria and were included, in addition to 25 healthy elderly subjects.

A fall history was present in 96 patients, syncope in six patients (of whom three patients also presented with additional falls) and dizziness/ near falling was present in six patients. The healthy elderly had no fall history nor history of syncope.

Tables 1 and 2 show the baseline characteristics and the Finapres test protocol results of patients and healthy elderly. There were more female than male patients, and more male than female healthy elderly.

**Table 1:** Baseline characteristics and Finapres protocol results of patients.

**Variable**	**Patients (n=105)**
Age (years)	78.7 (7)
Female	70 (67)
SBP* ‘clinic’ (mmHg)	161.6 (23.6)
DBP^†^ ‘clinic’ (mmHg)	83.3 (11.6)
Heart rate (beats/min)	67 (13)
CIRS-G^‡^	11.3 (4.7)
GARS^§^	30.2 (8.1)
Tinetti	21.3 (6.1)
Polypharmacy^¶^	68 (65)
Antihypertensive drugs	60 (57)
Stroke history	26 (25)
Diabetes mellitus	22 (21)
Parkinson’s disease	6 (6)
Dementia	6 (6)
SBP Finapres (mmHg)	169.1 (26.3)
Baroreflex index	0.28 (1.57)
Diagnosis OH^#^	50 (48)
Diagnosis PPH**	56 (53)
Diagnosis CSH^††^	61 (58)

Note. The values are the mean (SD) for normally distributed variables and n (percentages) for categorical variables. *SBP, systolic blood pressure; ^†^DBP, diastolic blood pressure; ^‡^CIRS-G; Cumulative Illness Rating Scale for Geriatrics; score 0-4, higher scores reflect more comorbidity, which reflects the severity of pathology in each of 14 categories (maximum score 56); ^§^GARS; possible range 0-53, higher score indicates greater impairment; ^¶^Polypharmacy was defined as the use of more than three drugs; ^#^OH, orthostatic hypotension; **PPH, postprandial hypotension; ^†^^†^CSH, carotid sinus hypersensitivity.

**Table 2:** Baseline characteristics of subjects undergoing negative and positive head-turning tests (HTT) in the healthy group.

**Variable**	**All (n=25)**	HTT-* (n=14)	**HTT+^†^ (n=11)**
Age (years)	74.7 (4.4)	75.6 (5.1)	73.6 (3.3)
Male	20 (80)	11 (79)	9 (82)
BMI^‡^ (kg/m^2^)	25.4 (2.2)	25.4 (2.1)	25.5 (2.5)
Lawton	8 (0)	8 (0)	8 (0)
SBP^§^ Finapres (mmHg)	143.1 (17.8)	137.1 (19.4)	150.6 (12.7)
Baroreflex index	0.8 (0.74)	1.05 (0.91)	0.50 (0.30)
Diagnosis OH^¶^	7 (25)	2 (14)	5 (36)
Diagnosis PPH^#^	16 (64)	8 (57)	8 (73)
Diagnosis CSH**	6 (24)	1 (7)	5 (45)
OH drop (mmHg)	38 (19)	50 (28)	34 (16)
PPH drop (mmHg)	35 (14)	36 (10)	34 (18)
CSH drop (mmHg)	65 (10)	74	63 (10)
HTIH^††^ drop (mmHg)	NA^‡‡^	NA	35 (19)

Note. The values are the mean (SD) for normally distributed variables and n (percentages) for categorical variables. *HTT-, negative head-turning test; ^†^HTT+, positive head-turning test; ^‡^BMI, body mass index; ^§^SBP, systolic blood pressure; ^¶^OH, orthostatic hypotension; ^#^PPH, postprandial hypotension; **CSH, carotid sinus hypersensitivity; ^†^^†^HTIH, head turning-induced hypotension; ^‡‡^NA, not applicable.

**Figure 1:**
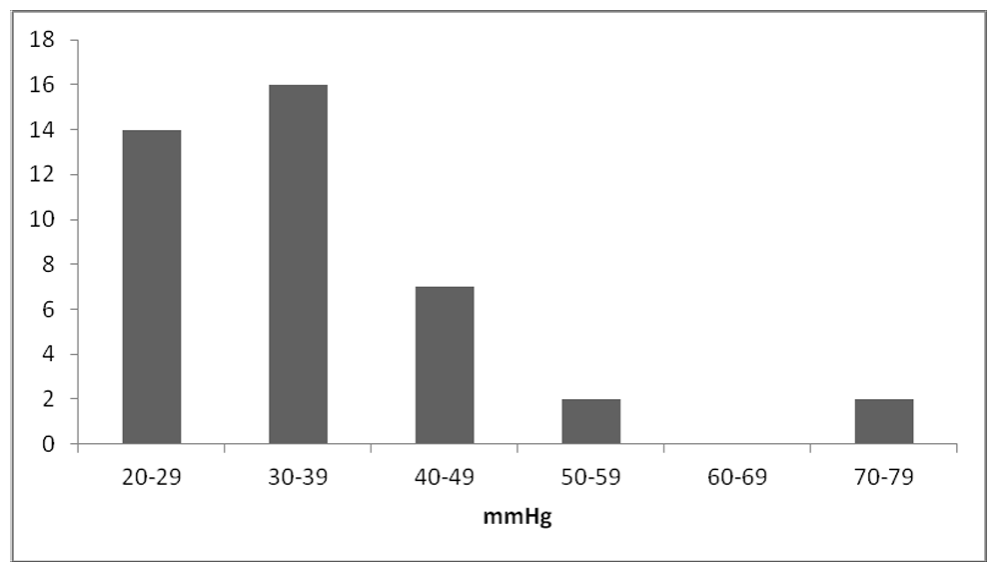
Distribution of maximal systolic blood pressure drops due to head rotation in the patient group. Notes. X-axis; the maximal systolic blood pressure drop due to head rotation, Y-axis; the number of patients that met the specified systolic blood pressure drop. The systolic blood pressure drops of 20-29 mmHg are due to left head rotation in 3 patients, right head rotation in 4 patients and hyperextension in 7 patients. The systolic blood pressure drops of 30-39 mmHg are due to left head rotation in 5 patients, right head rotation in 5 patients and hyperextension in 6 patients. The systolic blood pressure drops of 40-49 mmHg are due to left head rotation in 2 patients, right head rotation in 1 patients and hyperextension in 4 patients. The systolic blood pressure drops >50 mmHg are due to left head rotation in 4 patients.

**Figure 2:**
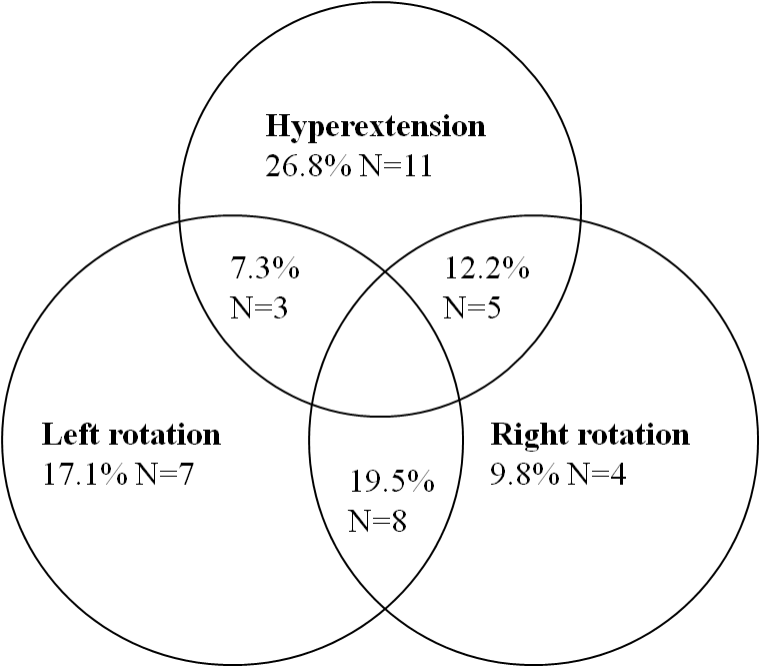
Venn diagram showing the distribution of head turning-induced hypotension across the different head movements.

Head turning led to hypotension in 39% of patients, with a mean systolic blood pressure drop of 36 mmHg (SD ±13; range 20-76) (Figure 1). A total of 26% patients had HTIH with a systolic blood pressure drop of 30 mmHg or greater, and there was a pressure drop of at least 50 mmHg in 4% of patients. Head turning led to hypotension irrespective of the direction of the head movement (Figure 2). HTIH was associated with a lower body mass index; p<0.01 [95%CI, 0.71 to 3.79], and a higher systolic blood pressure, both ‘clinic’; p<0.01 [CI, -20.62 to -2.25], and Finapres; p<0.01 [CI, -5.87 to -4.56].

Analysis of the data using a cut-off value of 30 mm Hg for HTIH did not change these results (Table 3).

**Table 3:** Baseline characteristics and results of negative and positive head-turning tests (HTT) in patients.

**Variable**	HTT-*	**HTT+^†^**	**P-value**	HTT-*	**HTT+^†^**	***P*-value**
	**(<20mmHg)**	**(≥20mmHg)**		**(<30mmHg)**	**(≥30mmHg)**	
	**(n=64)**	**(n=41)**		**(n=78)**	**(n=27)**	
Age (years)	78.2 (7.0)	79.8 (6.8)	0.26	78.3 (7.0)	80.1 (6.8)	0.25
Male	21 (32.8)	14 (34.1)	0.89	24 (30.7)	11 (40.7)	0.34
BMI^‡^ (kg/m^2^)	27.1 (4.1)	24.9 (3.5)	<0.01	26.9 (4.1)	24.4 (3.2)	<0.01
CIRS-G^§^	11.2 (4.9)	11.4 (4.5)	0.79	11 (4.8)	12 (4.5)	0.39
GARS^¶^	30.5 (8.5)	29.9 (7.5)	0.70	29.6 (8.3)	32.2 (7.4)	0.14
Polypharmacy^#^	42 (65.6)	26 (63.4)	0.82	49 (62.8)	19 (70.4)	0.48
Stroke history	12	14	0.08	16	10	0.09
Diabetes mellitus	14	8	0.77	15	7	0.47
Parkinson’s disease	3	3	0.58	4	2	0.66
Dementia	1	5	0.02	3	3	0.16
Hypertension	30 (46.9)	20 (48.8)	0.85	37 (47.4)	13 (48.1)	0.95
Antihypertensives	39 (60.9)	21 (51.2)	0.33	44 (56.4)	16 (59.3)	0.80
SBP** ‘clinic’ (mmHg)	157.1 (21.8)	168.6 (25)	0.02	158.5 (21.7)	170.7 (26.9)	0.02
DBP^††^ ‘clinic’ (mmHg)	82.4 (11.6)	84.5 (11.5)	0.37	82.7 (11.4)	84.8 (12.3)	0.43
Heart rate (beats/min)	66.8 (13.3)	67.4 (11.8)	0.82	67.1 (13)	67 (11.9)	0.97
SBP** Finapres (mmHg)	162.7 (24.1)	177.9 (29.4)	<0.01	163.9 (23.8)	182.7 (31.8)	<0.01
Baroreflex index	0.37 (1.87)	0.16 (1.03)	0.52	0.29 (1.84)	0.27 (0.16)	0.95
Diagnosis OH^‡‡^	27 (42.2)	23 (56.1)	0.16	33 (42.3)	17 (63)	0.06
Diagnosis PPH^§§^	35 (54.7)	21 (51.2)	0.73	43 (55.1)	13 (48.1)	0.53
Diagnosis CSH^¶¶^	30 (46.9)	31 (75.6)	<0.01	40 (51.3)	21 (77.8)	0.02

Note. The values are the mean (SD) for normally distributed variables and n (percentages) for categorical variables. *HTT-, negative head-turning test; ^†^HTT+, positive head-turning test; ^‡^BMI, body mass index; ^§^CIRS-G; Cumulative Illness Rating Scale for Geriatrics; score 0-4, higher scores reflect more comorbidity, which reflects the severity of pathology in each of 14 categories (maximum score 56); ^¶^GARS; possible range 0-53, higher score indicates greater impairment; ^#^Polypharmacy was defined as the use of more than three drugs; **SBP, systolic blood pressure; ^††^DBP, diastolic blood pressure; ^‡‡^OH, orthostatic hypotension; ^§§^PPH, postprandial hypotension; ^¶¶^CSH, carotid sinus hypersensitivity.

In healthy elderly, head turning also led to hypotension in 11 individuals (44%), with a mean systolic blood pressure drop of 35 mmHg (SD±19; range 20-85). This prevalence did not differ from patients (p=0.65).

Carotid sinus hypersensitivity was present in 61 patients, of whom half had hypotension that was induced by head-turning (Table 3). The prevalence of HTIH was higher in patients with CSH (51%) than without CSH (23%); the unadjusted odds ratio for the presence of HTIH in patients with CSH was 3.5 [CI, 1.48 to 8.35]. The adjusted odds ratio for the presence of HTIH in patients with CSH was 3.3 [CI, 1.28 to 8.36, adjusted for age, sex, body mass index and systolic blood pressure]. The subtypes of carotid sinus hypersensitivity were similarly distributed in patients with and without HTIH. There was no correlation between the maximal systolic blood pressure drop in HTIH and the maximal systolic blood pressure drop in CSH; r=0.26, p=0.10. Carotid sinus hypersensitivity was present in 6 healthy elderly, of whom 5 had a positive head-turning test.

During the head-turning test, 26 patients and one healthy control reported symptoms of dizziness or light-headedness, however, these symptoms were unrelated to the presence of HTIH (present in 9 out of these 26 patients) or carotid sinus hypersensitivity. Nine patients with HTIH had symptoms, but these symptoms were not related to the degree of systolic blood pressure decline; p=0.95 [CI, -9.9 to 10.5]. The question of whether a fall was ever preceded by shaving or head turning was answered affirmatively by 20 patients, but was not correlated with carotid sinus hypersensitivity; p=0.72, or HTIH; p=0.95.

Finally, we performed a subgroup analysis of patients for whom the physician suspected a hypotensive syndrome as the cause of falls (prior to knowing the results of the tests) versus those patients where hypotension was not thought to play a role. In 45 patients the physician had stated that he suspected an hypotensive syndrome; HTIH was present in 17 of these 45 patients, whereas HTIH was present in 13 patients out of 33 patients in whom the physician did not suspect hypotension (p=0.89).

## Discussion

The main finding of this study is that head turning triggers hypotension in nearly 40% of elderly patients referred for a fall or syncope. This head-turning induced hypotension appears to be a manifestation of carotid sinus hypersensitivity and can be added to the list of common hypotensive triggers in the elderly, including orthostatic and postprandial hypotension, as it is comparable both in prevalence and in its hypotensive effect [6]; [7].

The strength of this study is that the effect of head turning on blood pressure was investigated prospectively in consecutive patients irrespective of whether blood pressure instability (episodic hypotension) was suspected after clinical evaluation. Thus, patients with explanations for their fall other than hypotension were not excluded from this study. This approach reduced selection bias. Nonetheless, the study population was selective, as these patients were referred to a falls clinic for falls, dizziness and/or syncope. Therefore, the probability of finding hypotension was high and explains that more than half of this geriatric population met the criteria for orthostatic hypotension, postprandial hypotension or carotid sinus hypersensitivity. Nevertheless, in such a selected population there are also other evident explanations for falls [20]. Female patients were overrepresented and, as was illustrated by comorbidities, functional performance scores and polypharmacy, the patients can be regarded as frail. Indeed, a high prevalence of hypotensive syndromes is correlated with comorbidity [19]; [21]. This relationship limits the generalisation and external validity of our findings to the general elderly population. However, we tried to address this limitation by including a group of healthy elderly, recruited in the community, that was predominantly male, was not frail, and had no history of falls or syncope. Although this group was considerably smaller, their findings were very similar to those of the patient group, suggesting that the observation of HTIH is not a result of the selection of a specific set of patients, and reinforces the suggestion that the phenomenon is mechanistically related to carotid hypersensitivity.

In agreement with our hypothesis, there was an association between HTIH and CSH, namely patients with CSH were more likely to have HTIH than patients without CSH, and CSH was diagnosed in 75% of patients with HTIH. It was a priori not expected to find a 100% association between HTIH and CSH. First, both tests can reveal false-negative test results and second, the actual effect on the carotid sinus during CS massage versus HTT may be different; CS massage may cause increased pressure on the carotid sinus, while head turning may cause stretching of the carotid sinus. It is therefore more likely that CSH was missed in some of the subjects with HTIH than that explanations other than CSH explain the hypotensive effect of head turning in these subjects.

The pathophysiology of CSH remains debated [8]. The baroreflex sensitivity is reduced with ageing, i.e. the baroreflex response to blood pressure changes is reduced. In contrast, in CSH, the baroreflex response is exaggerated but is probably mediated by external stimuli to the baroreceptors, not by blood pressure per se. Head turning has been shown to increase cardiovagal baroreflex sensitivity, which could lower the threshold for carotid sinus sensitivity [22]. The high prevalence of CSH is thought to underlie the increasing prevalence of syncope with advancing age [23].

Alternative explanations for the hypotensive effect of head turning must be considered. Active head turning causes muscle activation and stimulation of the vestibular system. Activation of the neck afferents affects reflex autonomic cardiovascular control [24]. The vestibular system influences autonomic control and plays a role in postural-related adjustments to blood pressure [25]; [26]. This vestibulosympathetic reflex influences blood pressure by increasing muscle sympathetic nerve activity [25]; [26]. This reflex, however, attenuates with ageing [27]. Moreover, the effects on blood pressure are very small (well below 10 mmHg) and therefore this mechanism is unlikely to explain HTIH [27].

The subclavian steal syndrome can rarely lead to symptoms such as syncope, due to arteriosclerotic occlusion of the proximal subclavian artery. Although the physical examination findings of the subclavian steal syndrome includes a blood pressure difference of greater than 20 mmHg between arms, subclavian steal rarely induces a hypotensive episode. This only occurs if subclavian steal leads to brain stem hypoperfusion and thereby affects autonomic blood pressure control [28]. Likewise, carotid artery stenosis may cause neurologic symptoms such as a transient ischemic attack, but will not lead to hypotensive episodes.

HTIH was associated with a lower body mass index, a higher systolic blood pressure and a diagnosis of CSH. Analysis of the data using a cut-off value of 30 mm Hg for HTIH, which created a subgroup of patients with more severe hypotensive episodes during head turning, did not change these findings. The association between HTIH and body mass index might suggest that increased neck tissue fat reduces the impact of head turning on the carotid sinus [29].

The association between HTIH and high systolic blood pressure may be explained by the effect of elevated systolic pressure on the carotid wall and thereby on the carotid sinus stretch receptors involved in the baroreflex. This effect may predispose hypertensive elderly to CSH and HTIH.

Although systolic blood pressure decreased in HTIH, heart rate remained stable. This finding suggests that head turning is associated with vasodepression but not bradycardia. However, heart rate was only measured together with the blood pressure nadir, and slowing of the heart rate prior to this blood pressure nadir may have been missed.

A strength of our study is that our method of continuous blood pressure measurement generated many assessments of blood pressure, thereby increasing the method’s sensitivity to detect hypotension.

A drawback of the observational design is that it does not allow for conclusions about the causal relationships among HTIH and syncope, and falls.

There is a discrepancy between occurrence of HTIH and symptoms related to head turning, because HTIH was asymptomatic in the majority of patients. This finding is in agreement with findings in the established hypotensive syndromes (CSH, OH and PPH), which often fail to produce symptoms during measurements. In addition, these hypotensive syndromes are frequently found in healthy elderly subjects, as is also the case in this study. We found a similar occurrence of HTIH in patients and healthy elderly. We speculate that the appearance of symptoms during a hypotensive episode depends on the magnitude of the reduction, the total duration of the hypotensive period, and the capacity of cerebral autoregulation to restore cerebral blood flow before blood pressure is fully restored [16].

In conclusion, head turning may cause hypotensive episodes in the elderly. These data provide a plausible causal link to explain the well-established association between carotid sinus hypersensitivity and unexplained falls, and they provide evidence to support counselling patients with CSH about the effects of head rotation on blood pressure, with the advice to minimize extreme head turning.
